# Coherent consolidation of trillions of nucleations for mono-atom step-level flat surfaces

**DOI:** 10.1038/s41467-023-36301-w

**Published:** 2023-02-08

**Authors:** Taewoo Ha, Yu-Seong Seo, Teun-Teun Kim, Bipin Lamichhane, Young-Hoon Kim, Su Jae Kim, Yousil Lee, Jong Chan Kim, Sang Eon Park, Kyung Ik Sim, Jae Hoon Kim, Yong In Kim, Seon Je Kim, Hu Young Jeong, Young Hee Lee, Seong-Gon Kim, Young-Min Kim, Jungseek Hwang, Se-Young Jeong

**Affiliations:** 1 Center for Integrated Nanostructure Physics, Institute for Basic Science, Sungkyunkwan University, Suwon, 16419 Republic of Korea; 2 Department of Physics, Sungkyunkwan University, Suwon, 16419 Republic of Korea; 3 Department of Physics, University of Ulsan, Ulsan, 44610 Republic of Korea; 4 Department of Physics and Astronomy, Mississippi State University, Mississippi State, MS 39762 USA; 5 Department of Energy Science, Sungkyunkwan University, Suwon, 16419 Republic of Korea; 6 Crystal Bank Research Institute, Pusan National University, Busan, 46241 Republic of Korea; 7 School of Materials Science and Engineering, Ulsan National Institute of Science and Engineering, Ulsan, 44919 Republic of Korea; 8 Department of Physics, Yonsei University, Seoul, 03722 Republic of Korea; 9 UNIST Central Research Facilities, Ulsan National Institute of Science and Technology, Ulsan, 44919 Republic of Korea; 10 Department of Cogno-Mechatronics Engineering, Pusan National University, Busan, 46241 Republic of Korea; 11 Department of Optics and Mechatronics Engineering, Pusan National University, Busan, 46241 Republic of Korea

**Keywords:** Surfaces, interfaces and thin films, Structural properties

## Abstract

Constructing a mono-atom step-level ultra-flat material surface is challenging, especially for thin films, because it is prohibitively difficult for trillions of clusters to coherently merge. Even though a rough metal surface, as well as the scattering of carriers at grain boundaries, limits electron transport and obscures their intrinsic properties, the importance of the flat surface has not been emphasised sufficiently. In this study, we describe in detail the initial growth of copper thin films required for mono-atom step-level flat surfaces (MSFSs). Deposition using atomic sputtering epitaxy leads to the coherent merging of trillions of islands into a coplanar layer, eventually forming an MSFS, for which the key factor is suggested to be the individual deposition of single atoms. Theoretical calculations support that single sputtered atoms ensure the formation of highly aligned nanodroplets and help them to merge into a coplanar layer. The realisation of the ultra-flat surfaces is expected to greatly assist efforts to improve quantum behaviour by increasing the coherency of electrons.

## Abstract (web summary)

Constructing atomically flat surface in a single crystal ultrathin film is difficult owing to the coherent merging of trillions of clusters. Here the authors establish the initial growth mechanism of a single crystal Cu thin film with atomically flat surface using atomic sputtering epitaxy.

## Introduction

Ultrathin metal films are indispensable in modern electronics and nanotechnology^1–3^. During the past few decades, conventional metals have been studied extensively because the performance of metal-based devices is intimately related to their physical properties^4–6^. Efforts to produce single-crystal (SC) copper (Cu) from Cu foil have been driven by competition^7,8^ and interest in their nanocrystalline nature and potential applications in large two-dimensional (2D) components consisting of matrials such as graphene and hexagonal boron nitride (h-BN)^9–11^.

However, despite the importance of metal thin-film flatness, there have been few reports because it is challenging to control flatness. Since the contact between the metal electrode and the semiconductor material decisively affects the properties of electronic and optoelectronic devices,^12,13^ a flat metal surface is proposed as a good solution to reduce contact resistance. The motion of electrons without scattering at surfaces and grain boundaries can also affect the carrier transport properties^14^. Single-crystal Cu thin films (SCCFs) on sapphires have recently been reported, and the formation of twin boundaries (TBs) has been investigated intensively^15–17^. Two orientations (ORs) adjacent to a TB are rotated by a certain angle in-plane and satisfy the symmetry operation exactly, while two ORs adjacent to a grain boundary (GB) are tilted both in out-of-plane and in-plane directions. However, in view of electronic motion, a much clearer distinction between TB and GB must be followed by precise microscopic analysis. Twin boundaries are not strong electron scatterers,^18^ while electrons are scattered at GBs if they have an in-plane mistilt even by 1°, even if ORs adjacent to GBs are aligned almost perfectly along the out-of-plane direction. The number of TBs produced near the interface gradually decreases and may disappear as the position approaches the surface^15^. Nevertheless, for the grown thin film to lead to an ultra-smooth surface, it is necessary to discuss the initial growth process precisely. A study using scanning tunnelling microscopy demonstrated that nanocrystalline Cu films cannot be flat because valleys and ridges are created by out-of-plane grain rotation^19,20^. A recent study revealed that thin Cu films fabricated on four-inch-diameter wafers using atomic sputtering epitaxy (ASE) have atomically flat surfaces overall, with occasional mono-atomic step edges, and the crystal quality is maintained even after a few years^21^.

In this study, we describe in detail the initial growth phases of ultrathin Cu films prepared using ASE (“Methods” section and Supplementary Fig. 1a). Through transmission electron microscopy (TEM) observations, we identified the structural evolution from quasi-zero-dimensional (0D) nanodroplets to quasi-2D thin films in the initial growth stage. It was demonstrated experimentally and theoretically that the only way to consolidate the trillions of nucleations and to achieve a mono-atom step-level flat surface (MSFS) is by the individual deposition of single atoms when considering long-range period lattice mismatch between Cu and substrate Al_2_O_3_ and the formation of GB and TB.

## Results

### Growth of an ultrathin SCCF

**Fig. 1:**
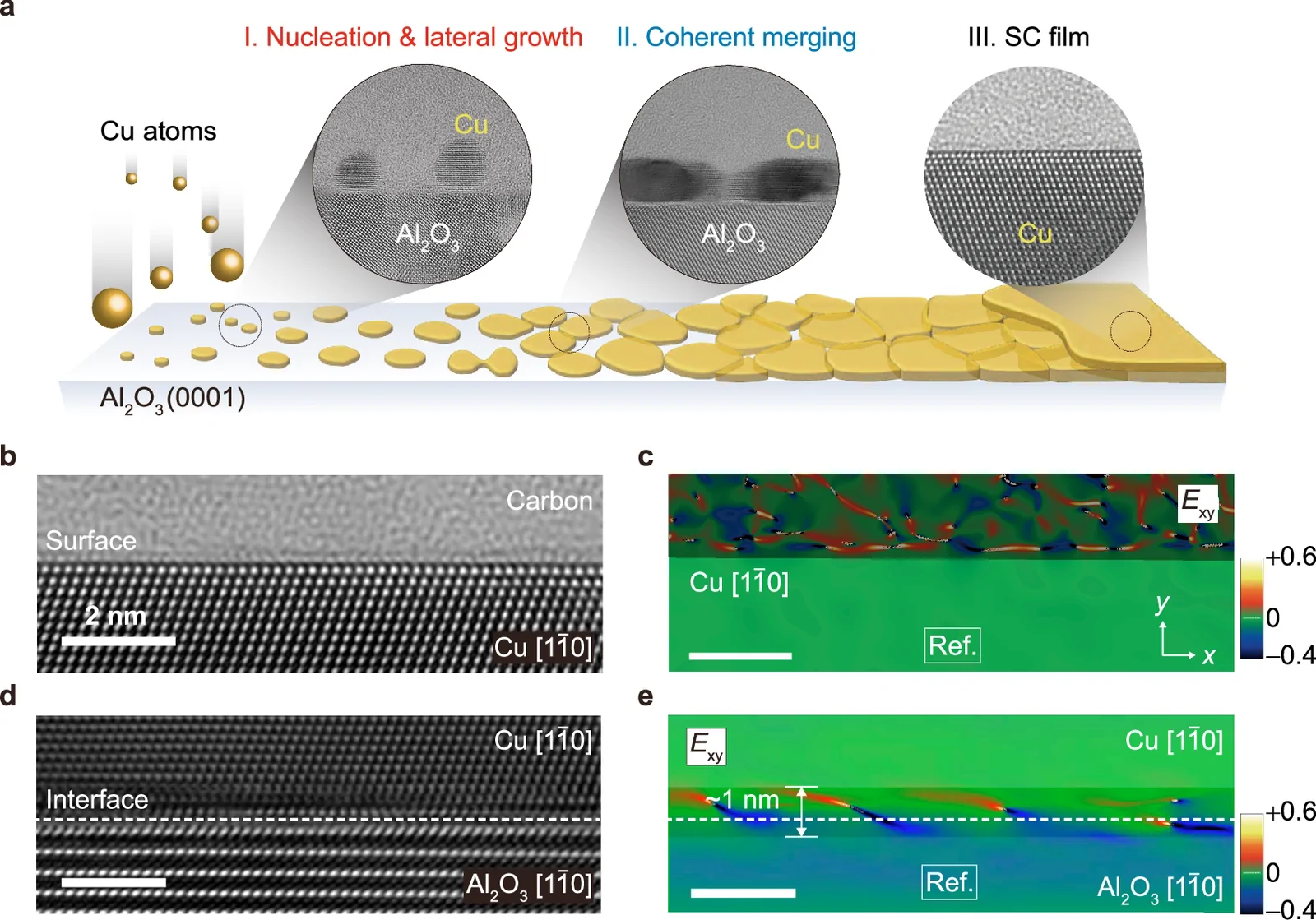
Coherent consolidation of nucleations and a mono-atom step-level flat surface. **a** Series of schematic diagrams of the structural evolution from quasi-zero-dimensional Cu(111) nanodroplets to a quasi-two-dimensional single-crystal Cu film in three stages: (I) nucleation and lateral growth, (II) coherent coplanar merging and (III) layer-by-layer growth. **b**, **c** Cross-sectional high-resolution transmission electron microscopy (HRTEM) image of a perfectly flat surface (left) and strain field map by geometrical phase analysis (GPA) (right), observed in the [1$\bar{1}$0] orientation. **d**, **e** Cross-sectional HRTEM image of the interfacial region of the Cu/Al_2_O_3_ heterostructure and its strain field map by GPA, with an orientation relationship of (111)_Cu_ [$1\bar{1}0$]_Cu_//(001)_Al2O3_[$1\bar{1}0$]_Al2O3_.

**Fig. 2:**
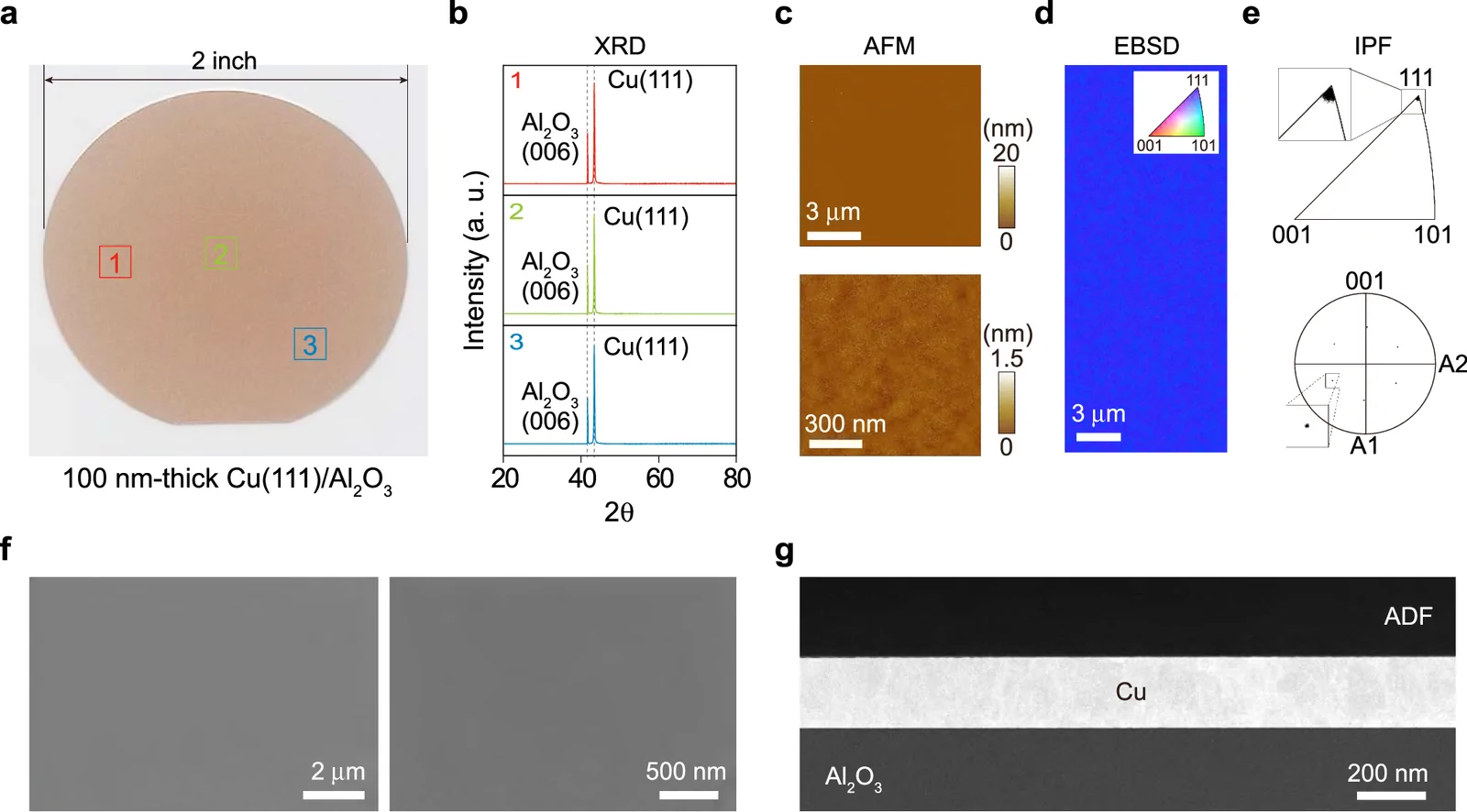
Single-crystal Cu film (SCCF) grown by atomic sputtering epitaxy (ASE). **a** Photograph of a 2-inch-diameter SCCF grown on Al_2_O_3_. **b** θ–2θ X-ray diffraction data taken at three different positions marked in **a**. **c** Surface morphologies at 20-nm (upper) and 1.5-nm (lower) vertical scales of atomic force microscopy (AFM) images with a root-mean-square surface roughness of 0.271 nm. **d** Electron backscatter diffraction (EBSD) map showing perfect alignment along the (111) plane. **e** Inverse pole figure (IPF) with a sole spot associated with the (111) plane (top) and [100] pole figure (PF) showing the six-fold symmetry of the {100} (bottom). The inset images in e are magnified images of the sole-spot areas. **f** Scanning electron microscopy images of the sample at different magnifications. **g** Low-magnification cross-sectional bright-field scanning transmission electron microscopy image of the SCCF sample.

Three approaches are typically used to grow thin films on a single-crystal substrate, i.e. the Volmer–Weber, Frank–van der Merwe and Stranski–Krastanov methods.^22^ In contrast, our ASE approach follows a new growth mode. The ideal initial growth of Cu on an Al_2_O_3_ substrate follows three stages (Fig. 1a): stage I, nanodroplet nucleation and lateral growth; stage II, coherent merging; and stage III, layer-by-layer growth of an SCCF. For the successful completion of this process, each of the trillions of nanodroplets must consist of a single crystal, and all must be aligned in the same direction. A high-resolution TEM image of a perfectly flat surface of a 12-nm-thick film and its strain field map obtained by geometrical phase analysis, respectively, are shown in Fig. 1b, c. The Cu thin films thicker than 11.5 nm are aligned perfectly along the (111) plane, which is supported by X-ray diffraction (XRD), atomic force microscopy (AFM), electron backscatter diffraction (EBSD) mapping, and scanning electron microscopy (SEM) and TEM images (Fig. 2a–g)^21^. The interface between Cu and the Al_2_O_3_ substrate after the growth of the 12-nm-thick film appears atomically abrupt and the Cu film above the strained interface region of about 1 nm in thickness grows defect- and strain-free (Fig. 1d, e).

### Coherent consolidation into a coplanar layer

**Fig. 3:**
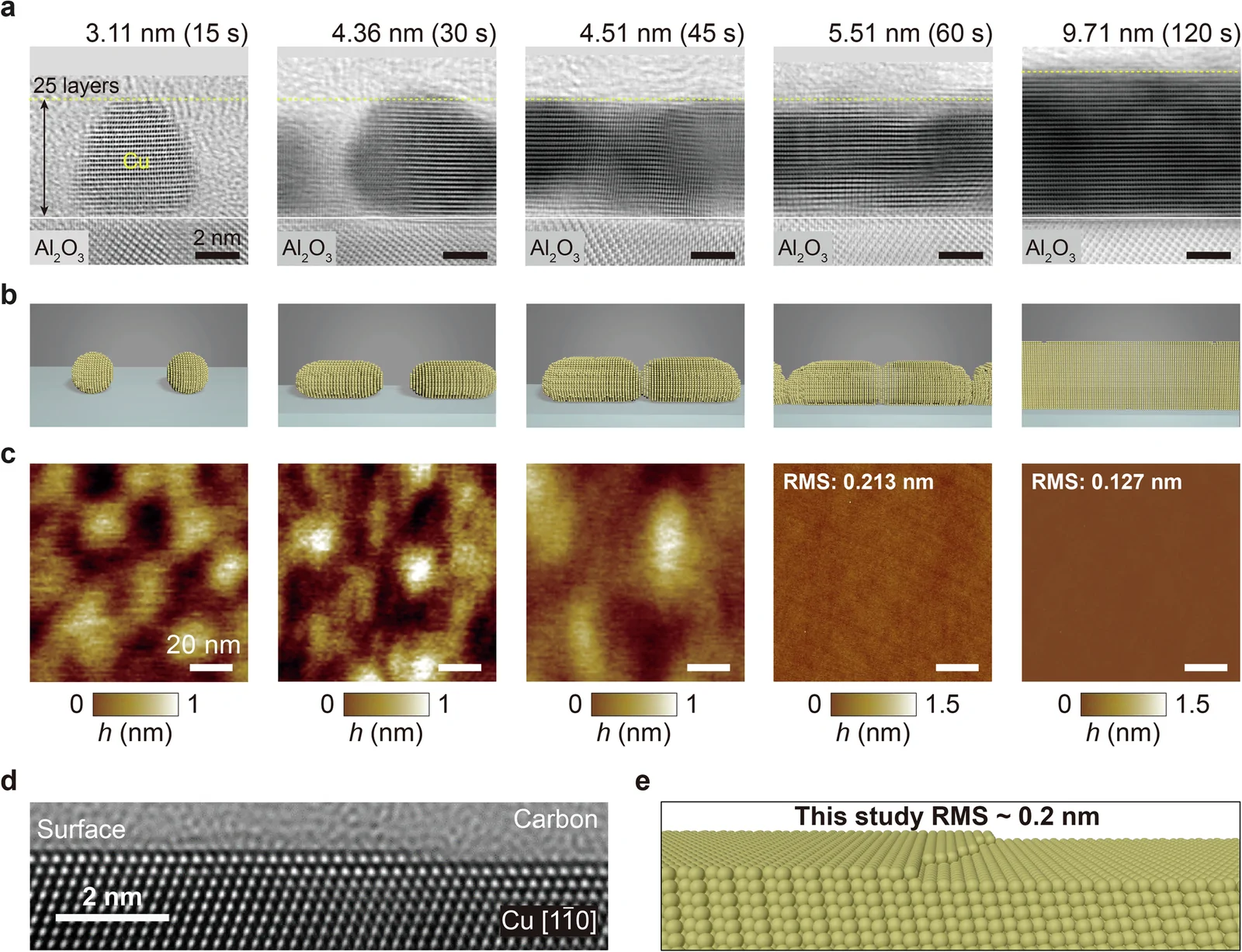
Coherent consolidation process for a mono-atom step-level flat surface. **a** Cross-sectional bright-field scanning transmission electron microscopy (BF STEM) images. **b** Corresponding illustrations. The island of twenty-five Cu layers (yellow dotted line in **a**, formed in the initial growth stage, is maintained and grows laterally. **c** Topographic atomic force microscopy images acquired from samples of five different thicknesses, corresponding to initial deposition times of 15, 30, 45, 60 and 120 s. **d** Cross-sectional high-resolution scanning transmission electron microscopy (HRTEM) image of the 12-nm-thick single-crystal Cu film showing mono-atom step-level flat surfaces (MSFS) with a root-mean-square (RMS) roughness of ~0.2 nm. **e** Schematic side view of MSFS in **d**.

The detailed states of early growth are shown in Fig. 3. Figure 3 shows a series of cross-sectional bright-field scanning TEM (BF-STEM) images^23^ acquired from samples of various thicknesses (Fig. 3a) along with corresponding illustrations (Fig. 3b) and topographic images obtained by atomic force microscopy (AFM; Fig. 3c).

Two separate initial stages are apparent in Fig. 3, divided according to a thickness threshold of ~5 nm. During stage I (Fig. 3a–c, first three panels), the Cu atoms form Cu(111) nanodroplets with single crystallinity; these nucleate at distances of 20–30 nm and consist of 18–31 layers (3–5 nm) on average, with a small height distribution (Supplementary Fig. 2). Because the area of an initial nanodroplet ranges from approximately 30 to 100 nm^2^, the total number of nanodroplets on a 2-inch-diameter wafer is approximately ~10^12^. During stage II, (Fig. 3a–c, fourth panels), Cu(111) nanodroplets begin to form conduction channels through Cu(111) lateral growth and coherent coplanar merging. The height of the nanodroplets increases more slowly (i.e. by a few layers) than the expected average deposition rate (i.e. ~7 layers per 15 s of initial deposition time), indicating predominant lateral growth. AFM images of <5-nm-thick films (Fig. 3c, first three images) obtained at a 1.0-nm resolution (for an area of 100 × 100 nm^2^) show the growth of islands separated by 20–30 nm, whereas thin films with a thickness of ≥5 nm (Fig. 3c, fourth image) obtained at a 1.5-nm resolution for a much larger area (10 × 10 µm^2^) exhibit a mainly flat surface.

Remarkably, thin films thicker than 10 nm showed an atomically flat surface with an exceptionally small root mean square (RMS) roughness of <0.2 nm. A thin film with RMS roughness of <0.3 nm has an atomically flat surface with occasional mono-atomic step edges. A cross-sectional HRTEM image (Fig. 3d) of 12 nm-thick Cu thin film and its graphical illustration (Fig. 3e) show the MSFS of the SCCF with an RMS roughness of ~0.2 nm. The fourth and fifth AFM images in Fig. 3c provide experimental evidence for the illustration in Fig. 3e and show that the thin film grows to an atomically flat surface beyond a thickness of ~10 nm. AFM images and RMS roughness as a function of thicknesses, EBSD and inverse pole figure (IPF), SEM images, and XRD data for 12 Cu films exhibiting marked Pendellösung oscillations (thickness fringes)^24^ also supported the quality of these films (Supplementary Fig. 3). The estimated thicknesses of all 12 thin-film samples obtained via AFM are listed in Supplementary Table 1. STM topography images show the MSFS on a large scale with single-atom step resolution (Supplementary Fig. 4).

**Fig. 4:**
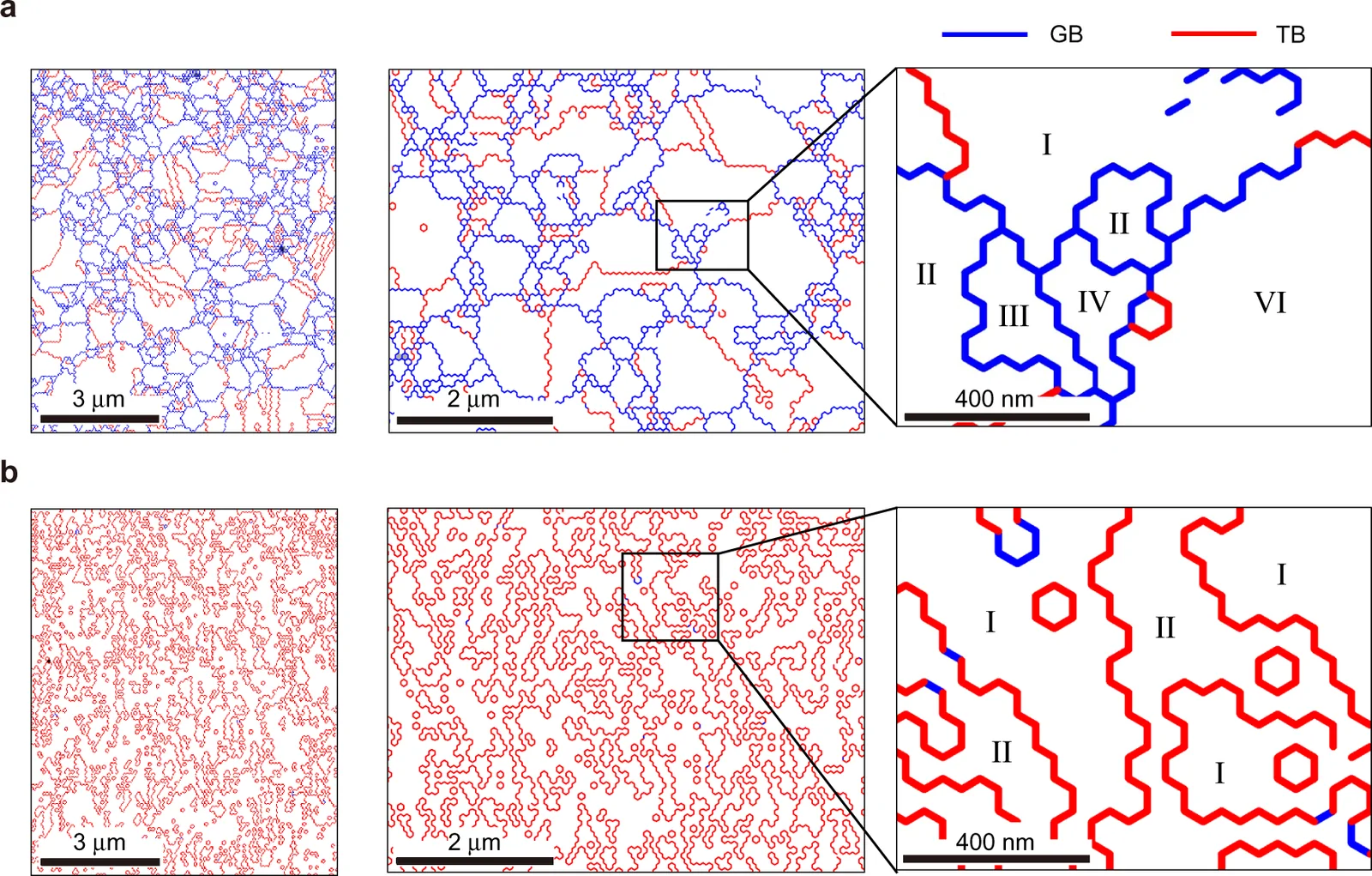
Grain boundaries (GBs) and twin boundaries (TBs). **a** Misorientation line distributions for a polycrystalline film. Red and blue lines denote TBs and GBs. Regions in the magnified image (third column) have various orientations (ORs). **b** Misorientation line distributions for a single-crystal Cu film. Misorientation lines are composed mostly of TBs. There are one of two ORs in the regions in the magnified image in the third column. The two regions separated by GBs in the polycrystalline film (**a**) are tilted at arbitrary angles both in the out-of-plane direction and in the in-plane direction. However, two adjacent regions in a single-crystal Cu film (**b**) are exactly 60° tilted in the in-plane direction, and both are completely parallel in the out-of-plane direction. The blue lines marked in **b** are classified as GB because they are slightly more tilted from 60° (<1°).

Twin boundaries or GBs in stage II critically affect the final roughness of the surface. The regions in a polycrystal have random ORs, which are separated mostly by GBs and sometimes by TBs (Fig. 4a), whereas regions in an SCCF have only two ORs and are separated by only TBs (Fig. 4b). The two ORs are associated with two different stacking orders, i.e. ABCABC··· and ACBACB···, and are separated by TBs with a closed path. Two different ORs in the SCCF must be rotated exactly by 60° to each other in-plane. The boundaries marked in blue (Fig. 4b) resemble TBs, but they are GBs because they have rotational components that deviate slightly from 60° in-plane^15^. Thus, it is not appropriate to identify TBs or GBs using an optical microscope or micrometre-scale EBSD map. Therefore, it is necessary to perform a misorientation line analysis at the nanoscale. Small ORs that are not observed at the micrometre scale are frequently observed at the nanometre scale. Two regions separated by a TB merge into a larger region of ~5–6 nm, and two enlarged regions double into a single OR at ~12 nm. With increasing thin-film thickness, the region of a single OR doubles every 5–6 nm similarly, and eventually (when the thickness reaches ~80 nm) the number of TBs in the upper part of the thin film is significantly reduced (Supplementary Fig. 5). Ideally, a single-crystal thin film, such as that grown homoepitaxially, can be obtained via heteroepitaxy near the surface when the thickness exceeds ~80 nm. Homoepitaxy-like heteroepitaxy of a Cu thin film on a hetero substrate is only possible when it is deposited by ASE, and the long-distance periodicity by the calculation of extended atomic distance mismatch^21^ is considered.

### Individual deposition of single atoms toward MSFS

**Fig. 5:**
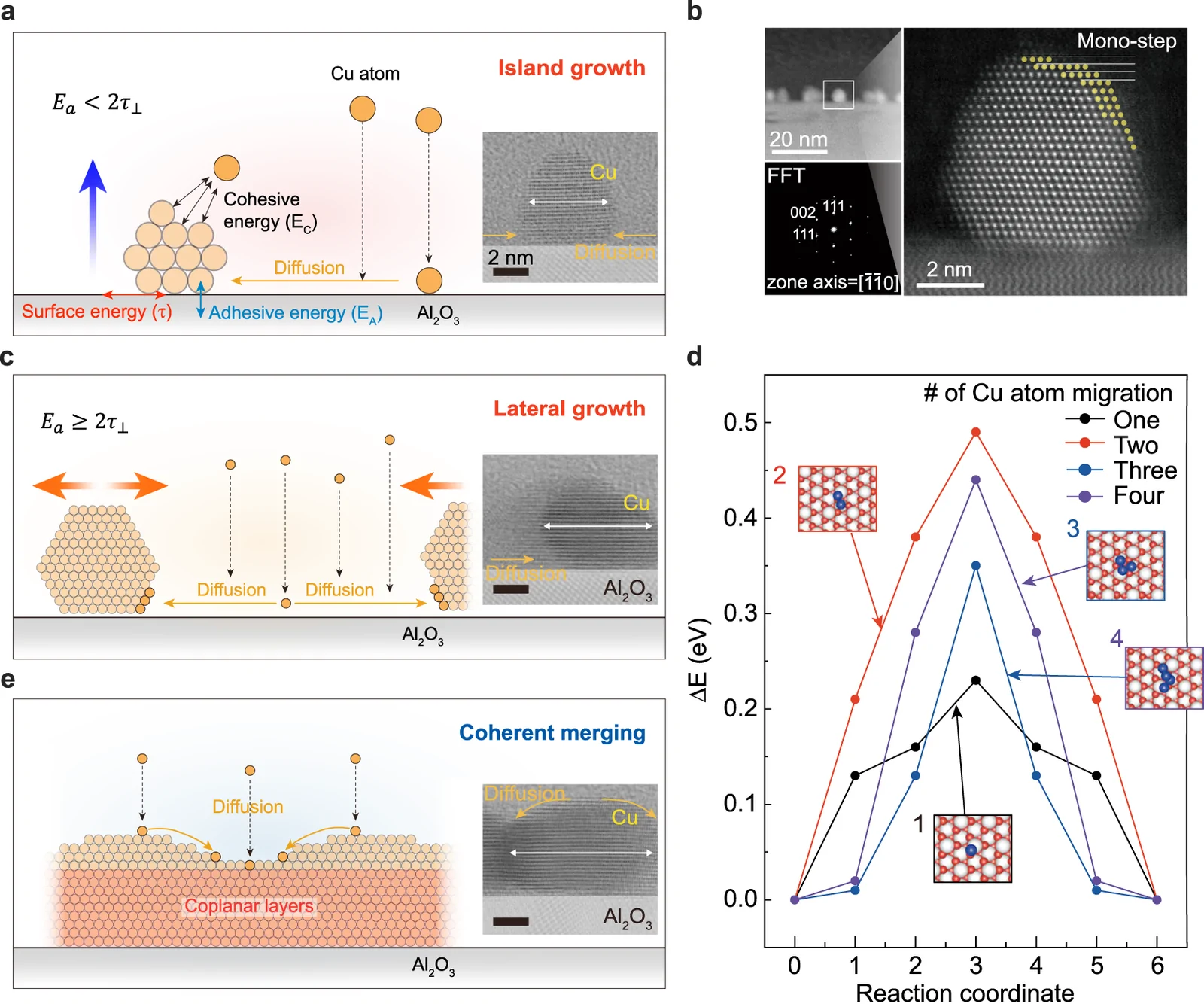
Theoretical approach for coherent consolidation of nucleations into a coplanar layer. **a** Scheme for the growth of a single nanodroplet via atomic diffusion, and corresponding bright field scanning transmission electron microscopy (BF STEM) image. **b** High-resolution scanning transmission electron microscopy (HRSTEM) image and fast Fourier transform pattern (inset) of a Cu nanodroplet oriented along the $[1\bar{1}0]$ zone axis, indicating single-crystal nanodroplet growth along the single-crystal [111] dire**c**tion. **c** Schemes for lateral nanodroplet growth and corresponding BF STEM image. **d** Relative energy profiles for the diffusion of Cu atoms or atom clusters (up to four atoms) on an Al_2_O_3_(0001) substrate. **e** Schemes for coherent merging to coplanar layers and corresponding BF STEM image.

Copper forms SC nanodroplets even during the early growth stage (<10 s) (Figs. 3a and 5a, b). In this study, the droplet surfaces were composed of well-defined crystallographic facets (Fig. 5b). The nanodroplet FFT patterns indicated that the structure consists of a single phase of Cu (Fig. 5b, inset). These nanodroplets grow along the [111] direction of the Al_2_O_3_(0001) substrate in one of two stacking orders (ABCABC… or ACBACB…). The height distribution of the nanodroplets was narrow; beyond a critical height, these droplets stop growing vertically and grow only laterally (Supplementary Fig. 2f). We used a simple model calculation to develop a new thin film growth mode that explains the conditions under which transition of the growth mechanism occurs (“Methods” section). Our model showed that the evolution of the thin film growth mechanism depends on the relative strength of the surface tension and adhesion energy. Initially, deposition of Cu atoms occurs on the Al-terminated surface of the Al_2_O_3_(0001) substrate, which has a much lower surface tension (1.59 J/m^2^) than the O-terminated surface (4.26 J/m^2^) (Supplementary Fig. 6a and Supplementary Table 2). The adhesion energy of the Al-terminated surface was 0.68 J/m^2^, which is lower than the surface tension of Cu(111) (${\tau }_{\perp }$ = 1.34 J/m^2^).^25^ Therefore, the requirement for a positive aspect ratio (${E}_{a} < {2\tau }_{\perp }$, Methods) was met, and droplets begin to grow spherically at a large aspect ratio (*R*) of 0.63 (stage I). As the droplet grows, its bottom side flattens, and O atoms are incorporated into the interface between the Cu droplet and Al_2_O_3_(0001) substrate surface because the interfacial energy of the O-terminated surface is lower than that of the Al-terminated surface (Supplementary Fig. 6b and Supplementary Table 2). The transition of the interface from the Al- to O-terminated surface of the Al_2_O_3_(0001) substrate increased adhesion to the substrate because the adhesion energy of the fully developed interface of Cu(111) on the O-terminated surface was 4.81 J/m^2^. When the adhesion became sufficiently strong (${E}_{a} > {2\tau }_{\perp }$; i.e., at the critical height), apparent vertical growth was halted (i.e., R→0), and only lateral growth occurred (stage II, Fig. 5c). For SCCFs, the critical height was found to be 5–6 nm (Supplementary Fig. 2f). Because all islands grew along the (111) direction in one of two possible ORs associated with stacking order, two nanodroplets with the same stacking sequence merged to a larger single-crystal droplet with a coplanar layer. However, when two nanodroplets with different ORs were merged, they became a larger droplet with a TB, which retains three-fold rotational symmetry.

The number of these separate nanodroplets depends on the diffusion rate of Cu atoms on the substrate. If the diffusion rate is high, then Cu atoms deposited on the substrate move over a large distance before coalescing with existing nanodroplets, forming fewer separate nanodroplet “islands”. If the diffusion rate is low, then the Cu atoms have a shorter range of motion, leading to the formation of more independent islands. The diffusion rate of the Cu atoms depends strongly on whether they are deposited on the substrate as single atoms or clusters of multiple atoms. The relative energy profiles of Cu atom diffusion onto an Al_2_O_3_(0001) substrate are shown in Fig. 5d, as obtained from first-principles calculations. The activation energies of clusters of multiple atoms were higher than those of single atoms; notably, the diffusion of clusters of two atoms required the most energy. This finding indicates that the deposition of single atoms during the early stage of nanodroplet nucleation is critical for the growth of an atomically flat SC thin film with uniform OR. Deposits of large clusters are likely to develop into a polycrystalline structure upon merging due to their random ORs. Although this requirement is challenging to fulfil in conventional sputtering systems, where Cu atoms are ejected and deposited as clusters of multiple atoms, the present ASE system meets this requirement.

To establish how much the sizes of the species falling off the target surfaces during the sputtering process differ between the conventional system and the ASE system, we compared the surfaces of two targets from the general sputtering system and the ASE system after the sputtering process (Supplementary Fig. 7). While the target surface of the general sputtering system was very rough, with an average RMS roughness of 100 nm (Supplementary Fig. 7a, c), the target surface of the ASE system had a smooth surface, with an average RMS roughness of 4 nm (Supplementary Fig. 7b, d). Optical images (left panels in Supplementary Fig. 7a, b) and AFM surface images at different scales for both targets (Supplementary Fig. 7c–f) showed critical differences. These results suggest that the difference in the size of the sputtered species during the sputtering process greatly influences thin film growth.

Once such a coplanar layer forms, the next layer is highly likely to grow in the same stacking order. After coherent merging, adhesion (with the layer of the adsorbate) became more dominant, and only layer growth occurred (stage III, Fig. 5e). Films thicker than 10 nm showed an ultra-flat, undistorted surface without multi-atomic step edges or grain boundaries (Fig. 1b).^26^ In a highly stable thin film growth system, where adsorbate atoms are deposited individually as single atoms, growth strictly follows the energetics of the surface tension and adhesion energy. The growth of GB-free homoepitaxy-like thin films with MSFS is not only possible for Cu, but also for other metals such as Ag.

### Initial growth stages

**Fig. 6:**
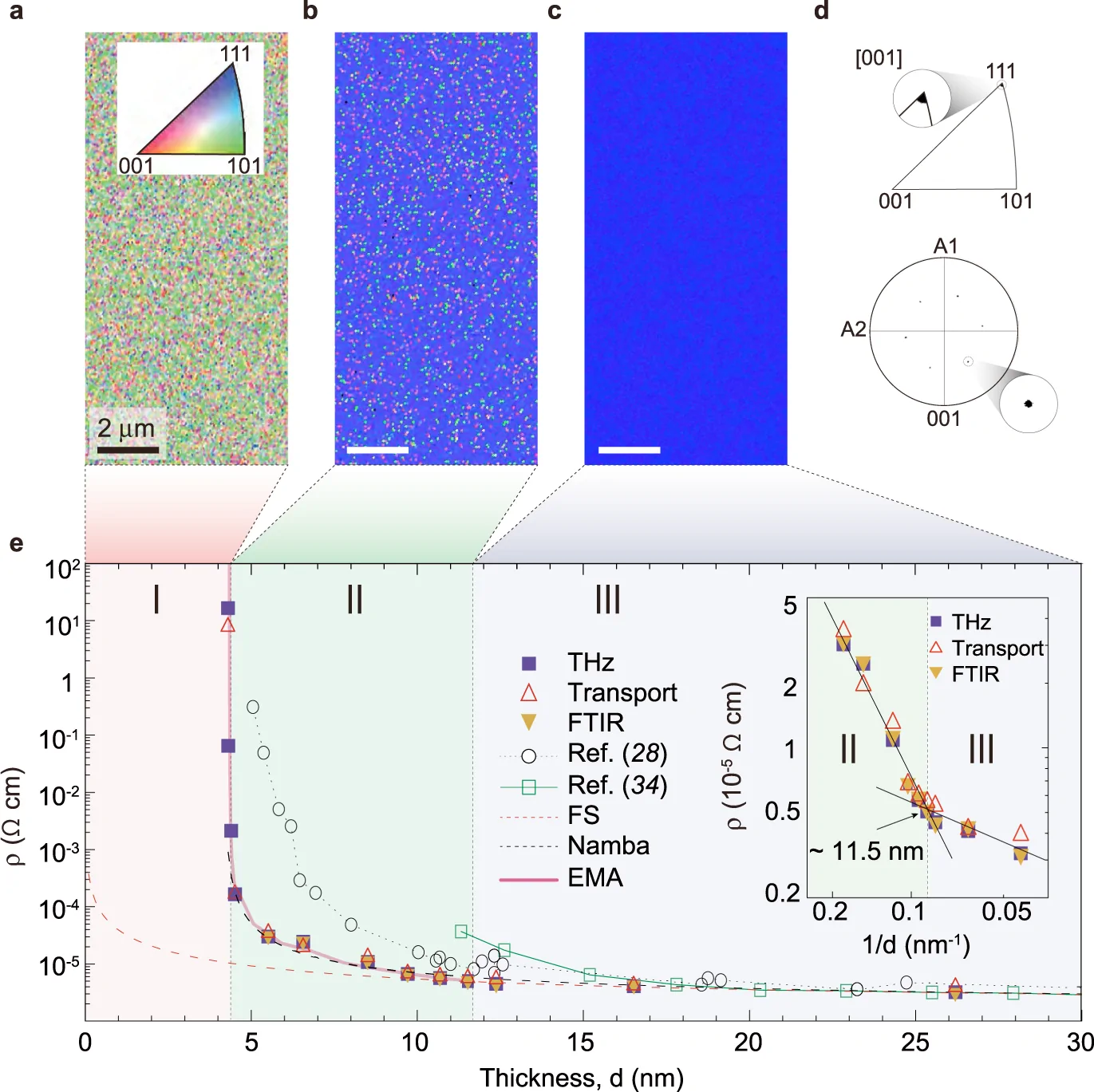
Three phases of the initial stages of film growth: nucleation, coherent merging and single-crystal thin film formation. **a–c** Electron backscatter diffraction images of Cu thin films in **a** stage I (Nucleation and island growth), **b** stage II (Coherent merging) and **c** stage III (SC film). **d** IPF with a sole spot associated with the (111) plane (upper panel) and [100] PF demonstrating the six-fold symmetry of the {100} PF (lower panel). The inset images in IPF and PF are enlarged images of the sole-spot areas. **e** Thickness-dependent resistivity (*ρ*) of SC films according to direct current electrical transport and optical Fourier transform-infrared spectroscopy measurements, as well as data from previous reports^28, 34^. Stages I and II are distinguished by the divergence of resistivity (or zero conductivity) at a film thickness (*d*) close to the nanodroplet size. Stages II and III are distinguished by the resistivity trend determined according to the resistivity slope change in *ρ* as a function of 1/*d* (inset).

Three distinct stages of initial film growth are also revealed by thickness-dependent DC resistivity (*ρ*) data (Fig. 6e) obtained by DC electrical transport,^27,28^ Fourier transform- infrared spectroscopy (FTIR) and time-domain terahertz spectroscopy (Supplementary Fig. 8). The transport data showed excellent agreement among the three methods. The DC resistivity (*ρ*) was close to the bulk value at thicknesses greater than ~11.5 nm, which was confirmed by plotting *ρ* as a function of the inverse of the film thickness (Fig. 6e, inset). Abrupt divergence near a thickness of ~4.6 nm indicated that the film was not conducting at lower thickness values. At thicknesses >~4.6 nm, the films had a finite *ρ*, indicating conduction channel formation. The results of analytical calculations using the Fuchs–Sondheimer (FS),^29,30^ Namba,^31^ and effective medium approximation (EMA)^32^ models are shown in Supplementary Fig. 9. The *ρ* values of films thicker than 11.5 nm were in good agreement with the FS model,^33^ whereas those of films thinner than 11.5 nm deviated from the FS model. The Namba model, which considers surface roughness, matched the data for surface roughness of ~4.5 nm, but could not explain *ρ* in stage I (<4.5 nm), in which Cu nanodroplets did not form conduction channels. The EMA method, which correctly predicts percolation for spherical grains, also described stages I and II well. The results indicate that the film does not occupy the full volume from the substrate surface to the film thickness during the initial growth stage. No reliable EBSD maps were obtained from <5-nm-thick films (i.e., stage I) (Fig. 6a), whereas thin films showed partial coverage at thicknesses between 5 and 11.5 nm (Fig. 6b) and complete coverage at > 11.5 nm (Fig. 6c). The EBSD map shown in Fig. 6c is indistinguishable from primary blue on the red–green–blue colour scale (0:0:255), indicating exact alignment along (111) with IPF and PF (Fig. 6d). We anticipate that ultrathin SCCFs will be employed in a wide range of high-technology applications. For example, we introduce ultrathin transparent Cu honeycomb mesh electrodes (Supplementary Fig. 10).

In summary, we implemented heteroepitaxy-like homoepitaxy using the sputtering system and grew thin films with MSFS. The critical requirement here is that each atom must be individually deposited during the sputtering process. Then, a myriad of nanodroplets merges through coherent lateral growth into coplanar layers at 11.5 nm, which is the critical thickness of a complete thin film. The thin film evolves to have an MSFS with an RMS roughness of <0.3 nm. In heteroepitaxy, the formation of TBs is inevitable but does not affect the formation of MSFS, whereas GBs have a strong effect. The individual deposition with single atoms enables the growth of thin films with MSFS not only for Cu but also for other metals, such as Ag.

## Methods

### Preparation of thin SCCF using the ASE technique

The network of conducting wires, including cables, in the conventional sputtering system, was replaced with SC Cu wires fabricated by cutting SC Cu wafers in a spiral fashion using a wire electrical discharge machining (wire-EDM). The Cu wafers were sliced from an SC ingot grown using the Czochralski method^35^. In our setup, vibration caused by ambient noise was minimised as much as possible using a mechanical noise reduction system. Although minute vibrations appear not to cause significant degradation in conventional thin film growth, especially for PCCFs, such miniscule mechanical vibrations can cause irreversible stacking faults that could significantly disturb the initial nucleation and lateral growth processes, especially the coherent coplanar merging of nuclei. However, the present ASE system provides a stable environment for single-atom deposition with the objective of achieving atomically flat surfaces through the stacking of single atoms. The optimised sputtering conditions using the ASE system are as follows. A double-sided polished (001) Al_2_O_3_ wafer with a thickness of 430 μm was used as the substrate. The deposition temperature and RF (13.56 MHz) power were approximately 170 °C and 30 W, respectively. The target-to-substrate distance was set at 95 mm. The base pressure was maintained at less than 2 × 10^−7^ Torr and the working pressure at 5.4 × 10^−3^ Torr with an Ar gas (99.9999% (6 N)) flow of 50 sccm. The relationship between the deposition time and thickness of the thin film (or the average growth rate) was determined from the average deposition time of a 200-nm-thick film grown under optimal conditions.

#### Structural information

For high-resolution (scanning) transmission electron microscopy [HR(S)TEM] analysis, a series of Cu samples grown on Al_2_O_3_(0001) substrates at deposition times ranging from 15 to 120 s were cross-sectioned for HR(S)TEM imaging using dual-beam focused ion beam (FIB) slicing (Helios NanoLab 450; FEI Co., Hillsboro, OR, USA) and lift-out processes. Double caesium (Cs)-corrected TEM (JEM-ARM200F; JEOL Ltd., Tokyo, Japan) at 200 kV was used to obtain BF-STEM and HRTEM images of the Cu samples. For high-resolution STEM imaging, the probe-forming semi-angle was 23 mrad. Statistically random background noise in the HR(S)TEM images was reduced using the 2D difference filtering method of a commercial software program (HREM-Filters Pro; HREM Research Inc., Tokyo, Japan). Chemical analysis of the Cu samples was performed using electron energy loss spectroscopy (EELS) with a post-column-type electron energy loss spectrometry system (GIF Quantum ER 965; Gatan, Pleasanton, CA, USA) equipped with the microscope.

For structural characterisation, XRD measurements were performed using a PANalytical Empyrean Series 2 diffractometer (Malvern PANalytical, Malvern, UK) with a Cu-Kα source (40 kV, 30 mA). Data were collected in the range of 20° <2θ < 90°, with a step size of 0.0167° and dwell time of 0.5 s per point in all cases. EBSD measurements were performed to confirm the quality of the thin films. A SUPRA40 VP SEM (Zeiss, Oberkochen, Germany) was used to measure EBSD maps of the thin films. The EBSD maps, pole figures (PFs), and IPFs show the directions and distributions of the crystals within the films. Surface roughness and sample thickness were measured by AFM using an XE-100 instrument (Park Systems, Suwon-si, South Korea). The basic scanning conditions included noncontact mode with an ~0.5 Hz scan rate and 1,024 × 1,024 resolution. The sample thickness was determined from an AFM image of Cu film scraped off using a sharp material. The scan rate was reduced to compensate for the stepped shape. The height resolution of the AFM instrument was 1.8 (0.25) Å in high-(low-)-voltage mode.

#### Transport and optical characteristics of SCCFs

For transport measurements, sheet resistance (Rs: Ω/sq) was measured in the van der Pauw geometry with an HMS-3000 Hall measurement system (Ecopia, Toronto, ON, Canada) under a 0.55-T magnetic field at room temperature. Resistivity data (*ρ*: Ω·cm) were obtained by multiplying sheet resistance by film thickness measured via AFM.

We investigated the optical quality of the ultrathin SCCFs using both infrared/optical spectroscopy and time-domain THz techniques. We measured the transmittance spectra of 12 Cu films of thicknesses ranging from 3 to 30 nm at room temperature. To conduct measurements over a wide spectral range from THz to UV wavelengths, we used a commercial THz spectrometer (TERA K15; Menlo, Planegg, Germany) for the spectral range below 100 cm^−1^, a commercial FTIR-type spectrometer (Vertex 80 v; Bruker, Karlsruhe, Germany) for the spectral range of 400–25,000 cm^−1^, and a commercial monochromatic spectrometer (Lambda 950; PerkinElmer, Waltham, MA, USA) for the spectral range from 3000 to 50,000 cm^−1^. Because the sapphire substrate has strong infrared-active phonons between 150 and 1500 cm^−1^, we could not obtain reliable spectra in this spectral range. However, no meaningful optical features of Cu films lie in this spectral range.

#### Theoretical approach

All ab initio total-energy calculations and geometry optimisations were performed using density functional theory (DFT) in the generalised gradient approximation (GGA) based on the Perdew–Burke–Ernzerhof functional^36^ and projected augmented plane-wave method,^37^ as implemented by Kresse et al.^38^ The Al_2_O_3_(0001) substrate was represented by a slab of 36 atomic layers of primitive unit cells containing 12 formula units, and the Cu thin film was represented by a slab of six layers of Cu atoms. The calculated lattice constants for bulk Al_2_O_3_ are *a* = 4.785 Å and *c* = 13.06 Å, in good agreement with the experimental values.^39^ A vacuum length of 15 Å was used, the bottom nine layers of the slab were fixed in their bulk positions, and the remaining atoms were fully relaxed until the Hellmann–Feynman force on each atom was <0.001 eV/Å and the change in total energy was <1 × 10^−5^ eV. A supercell containing a slab of 3 × 2 surface unit cells was used to simulate diffusion, and a slab of 1 × 1 surface unit cells was used to calculate the adhesion energy of Cu(111) on an Al_2_O_3_(0001) substrate. The electron wave functions were expanded in a plane-wave basis set with a cut-off energy of 420 eV, and Brillouin-zone integration for the slabs was performed using a 5 × 5 × 1 Monkhorst–Pack *k*-point grid.^40^ The nudged elastic band method^41^ was used to calculate the activation energy of diffusion with 0.01 eV/Å of the force criterion for structure optimisation.

We modelled the growth of nanodroplets into a thin film on a substrate by considering the total energy *E* of the droplet on a substrate, as follows:

1 $$E={-E}_{c}V+\int \tau {dS}-\int {E}_{a}d{S}_{a},$$

where *E*_*c*_ and τ are the cohesive energy per unit volume and surface tension, respectively, of a droplet with volume *V* and surface area *S*, and *E*_*a*_ is the adhesive energy per unit area of the interface between the droplet and a substrate with area *S*_*a*_. To simplify the analysis, we assumed that the droplet was a cylinder of radius *a* and height *h*. The surface energy term can be written as the sum of two terms: $\int \tau {dS}=2{\tau }_{\perp }{S}_{\perp }+{\tau }_{\parallel }{S}_{\parallel }$, where ${\tau }_{\perp }$ (${\tau }_{\parallel }$) is the average surface tension of the top (side) face of the droplet and ${S}_{\perp }=\pi {a}^{2}$ (${S}_{\parallel }=2\pi {ah}$) is the area of the top (side) face. To determine the shape of the droplet for a given amount (volume) of adsorbate atoms, we expressed the total energy in terms of the droplet height *h* and volume *V* using $a=\sqrt{V/\pi }{h}^{-1/2}$, as follows:

2 $$E=-{E}_{c}V+\left(2{\tau }_{\perp }-{E}_{a}\right)\pi {\left(\sqrt{V/\pi }{h}^{-1/2}\right)}^{2}+{\tau }_{\parallel }2\pi \left(\sqrt{V/\pi }{h}^{-1/2}\right)h.$$

The sign conventions were selected such that all physical quantities were positive for typical substrates and adsorbates. For a given droplet of volume *V*,

$$\frac{\partial E}{\partial h}=\left(2{\tau }_{\perp }-{E}_{a}\right)V\left(-{h}^{-2}\right)+{\tau }_{\parallel }2\sqrt{\pi }\sqrt{V}\left(\frac{1}{2}{h}^{-1/2}\right).$$

*E* is at its minimum when $\frac{\partial E}{\partial h}=0$:

3 $${\tau }_{\parallel }\sqrt{\pi }\sqrt{V}{h}^{3/2}=\left(2{\tau }_{\perp }-{E}_{a}\right)V.$$

When ${E}_{a} < {2\tau }_{\perp }$, the ratio between the height and lateral size is:

$$\frac{h}{a}=2\frac{{\tau }_{\perp }-{E}_{a}/2}{{\tau }_{\parallel }}.$$

Generalising this result to droplets of different shapes, and noting that 2*a* represents the lateral size, the evolution of the droplet shape can be expressed in terms of the aspect ratio *R* = *h*/2*a*, as follows:

4 $$\begin{array}{cc}R=f\frac{{\tau }_{\perp }-{E}_{a}/2}{{\tau }_{\parallel }} & {{{{\rm{for}}}}}\,{E}_{a} < {2\tau }_{\perp }\end{array}$$

Where *f* is a form factor on the order of unity that depends on the specific shape of the droplet.

If ${E}_{a}\ge {2\tau }_{\perp }$, the energy of the droplet in Eq. (2) is a monotonically increasing function of droplet height *h*, and minimising the energy causes *h* to approach zero (or *a* to increase limitlessly). Therefore,

5 $$\begin{array}{cc}R\to 0 & {{{{\rm{for}}}}}\,{E}_{a}\ge {2\tau }_{\perp },\end{array}$$

which is interpreted as a lack of droplet formation, with only lateral growth occurring.

## Supplementary information

Supplementary information

## Data Availability

The authors declare that the main data supporting the findings of this study are available within the article and its Supplementary Information files.
