# Author Correction: Coherent consolidation of trillions of nucleations for mono-atom step-level flat surfaces

**DOI:** 10.1038/s41467-026-76883-9

**Published:** 2026-08-20

**Authors:** Taewoo Ha, Yu-Seong Seo, Teun-Teun Kim, Bipin Lamichhane, Young-Hoon Kim, Su Jae Kim, Yousil Lee, Jong Chan Kim, Sang Eon Park, Kyung Ik Sim, Jae Hoon Kim, Yong In Kim, Seon Je Kim, Hu Young Jeong, Young Hee Lee, Seong-Gon Kim, Young-Min Kim, Jungseek Hwang, Se-Young Jeong

**Affiliations:** 1 Center for Integrated Nanostructure Physics, Institute for Basic Science, Sungkyunkwan University, Suwon, 16419 Republic of Korea; 2 Department of Physics, Sungkyunkwan University, Suwon, 16419 Republic of Korea; 3 Department of Physics, University of Ulsan, Ulsan, 44610 Republic of Korea; 4 Department of Physics and Astronomy, Mississippi State University, Mississippi State, MS 39762 USA; 5 Department of Energy Science, Sungkyunkwan University, Suwon, 16419 Republic of Korea; 6 Crystal Bank Research Institute, Pusan National University, Busan, 46241 Republic of Korea; 7 School of Materials Science and Engineering, Ulsan National Institute of Science and Engineering, Ulsan, 44919 Republic of Korea; 8 Department of Physics, Yonsei University, Seoul, 03722 Republic of Korea; 9 UNIST Central Research Facilities, Ulsan National Institute of Science and Technology, Ulsan, 44919 Republic of Korea; 10 Department of Cogno-Mechatronics Engineering, Pusan National University, Busan, 46241 Republic of Korea; 11 Department of Optics and Mechatronics Engineering, Pusan National University, Busan, 46241 Republic of Korea

**Keywords:** Surfaces, interfaces and thin films, Structural properties

Correction to: *Nature Communications* 10.1038/s41467-023-36301-w, published online 08 February 2023

In the version of this article initially published, in Fig. 3a, the scale bar units now reading “2 nm” appeared originally as “2 μm”; the figure is now amended in the HTML and PDF versions of the article.

