# Supplementary material for: Coherent consolidation of trillions of nucleations for mono-atom step-level flat surfaces
Source: Nat Commun. 2023 Feb 8;14:685. doi: 10.1038/s41467-023-36301-w (PMC9908865; doi:10.1038/s41467-023-36301-w)
Supplement: Supplementary file 1 — Supplementary information [file 41467_2023_36301_MOESM1_ESM.pdf]

Supplementary information

Coherent consolidation of trillions of nucleations for mono-atom step-level flat surfaces

Taewoo Ha[1†], Yu-Seong Seo[2†], Teun-Teun Kim[3†], Bipin Lamichhane[4], Young-Hoon Kim[5], Su Jae Kim[6], Yousil Lee[6], Jong Chan Kim[7], Sang Eon Park[6], Kyung Ik Sim[1,8], Jae Hoon Kim[8], Yong In Kim[5], Seon Je Kim[5], Hu Young Jeong[7,9], Young Hee Lee[1,5], Seong-Gon Kim[4], Young-Min Kim[1,5,*], Jungseek Hwang[2,*] & Se-Young Jeong[10,11,*]

[1]Center for Integrated Nanostructure Physics, Institute for Basic Science, Sungkyunkwan University, Suwon 16419, Republic of Korea.

[2]Department of Physics, Sungkyunkwan University, Suwon 16419, Republic of Korea.

[3]Department of Physics, University of Ulsan, Ulsan 44610, Republic of Korea.

[4]Department of Physics and Astronomy, Mississippi State University, Mississippi State, MS 39762, USA.

[5]Department of Energy Science, Sungkyunkwan University, Suwon 16419, Republic of Korea.

[6]Crystal Bank Research Institute, Pusan National University, Busan 46241, Republic of Korea.

[7]School of Materials Science and Engineering, Ulsan National Institute of Science and Engineering, Ulsan 44919, Republic of Korea.

[8]Department of Physics, Yonsei University, Seoul 03722, Republic of Korea.

[9]UNIST Central Research Facilities, Ulsan National Institute of Science and Technology, Ulsan 44919, Republic of Korea.

[10]Department of Cogno-Mechatronics Engineering, Pusan National University, Busan 46241, Republic of Korea.

[11]Department of Optics and Mechatronics Engineering, Pusan National University, Busan 46241, Republic of Korea.

*Corresponding authors.

E-mail: sk162@msstate.edu (S.-G. Kim); youngmk@skku.edu (Y.-M. Kim); jungseek@skku.edu (J. Hwang); syjeong@pusan.ac.kr (S.-Y. Jeong)

† These authors contributed equally to this work.

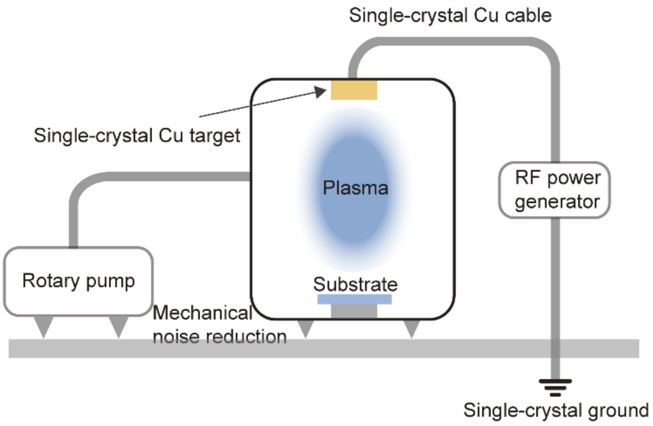

Supplementary Fig. 1. Schematic illustration of the atomic sputtering epitaxy (ASE) system. a, Schematic illustration of the ASE system for the mono-atom step-level flat surface. The three key factors ensuring individual deposition of single atoms are as follows: single-crystal Cu sputtering target, single-crystal power cable, and electrical internal wiring and grounding using single-crystal wires and a mechanical noise-reduction system using a mechanical diode structure.

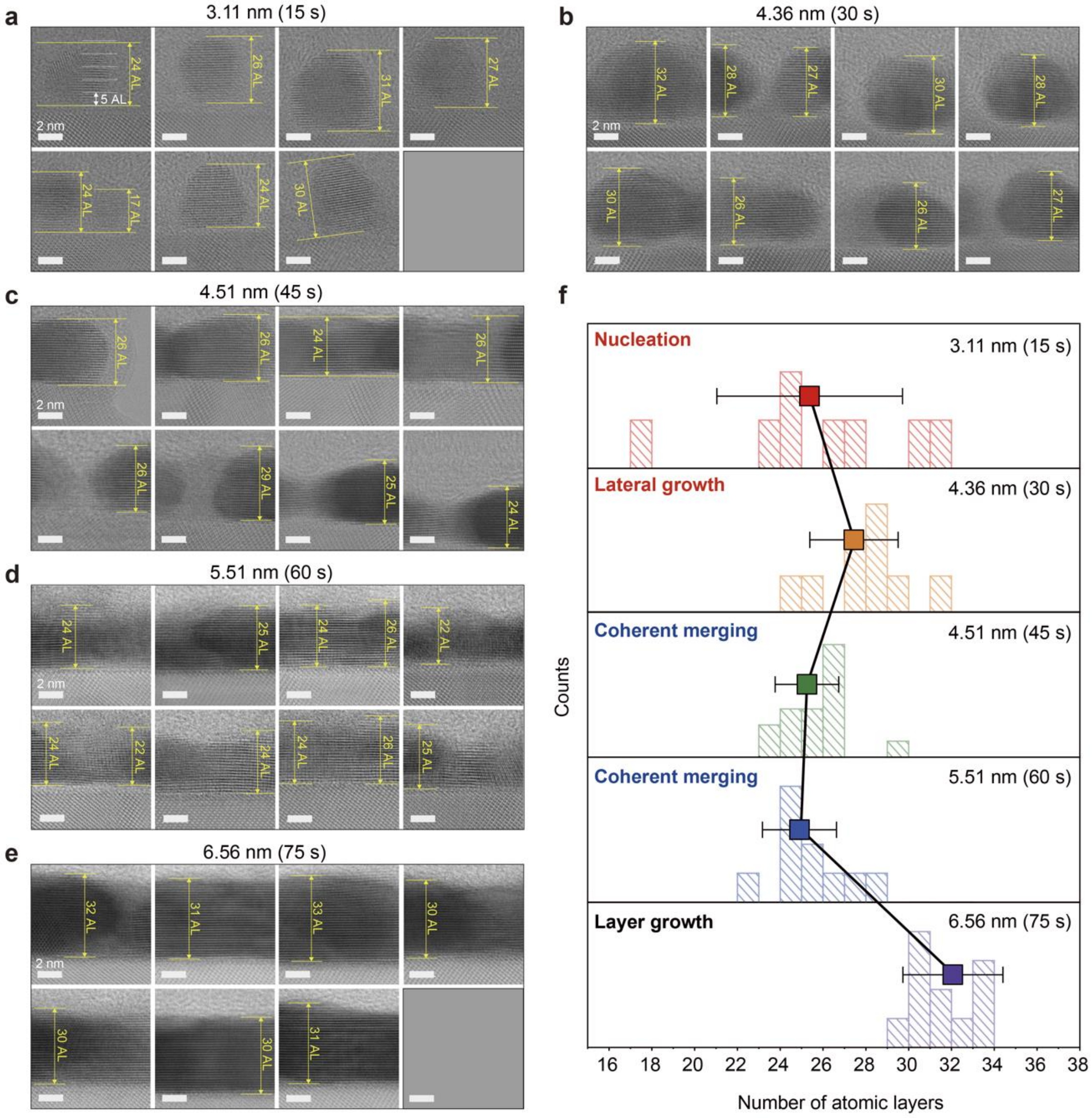

Supplementary Fig. 2. Height distribution of single-crystal Cu(111) nucleation nanodroplets in the first two stages: I (15, 30 and 45 s) and II (60 and 75 s). a–e, Bright-field scanning transmission electron microscopy images of thin films and average thickness of each film, as measured by atomic force microscopy. **a**, 3.11 nm (15 s), **b**, 4.36 nm (30 s), **c**, 4.51 nm (45 s), **d**, 5.51 nm (60 s) and **e**, 6.56 nm (75 s). Numbers in parentheses are the deposition times of the films. A full list of the measured thicknesses as a function of deposition time is provided in Supplementary Table S1. **f**, Histogram and average numbers of atomic layers (height) for each deposition time (15–75 s) with error bars through standard deviation of the counting layer in each STM image**.**

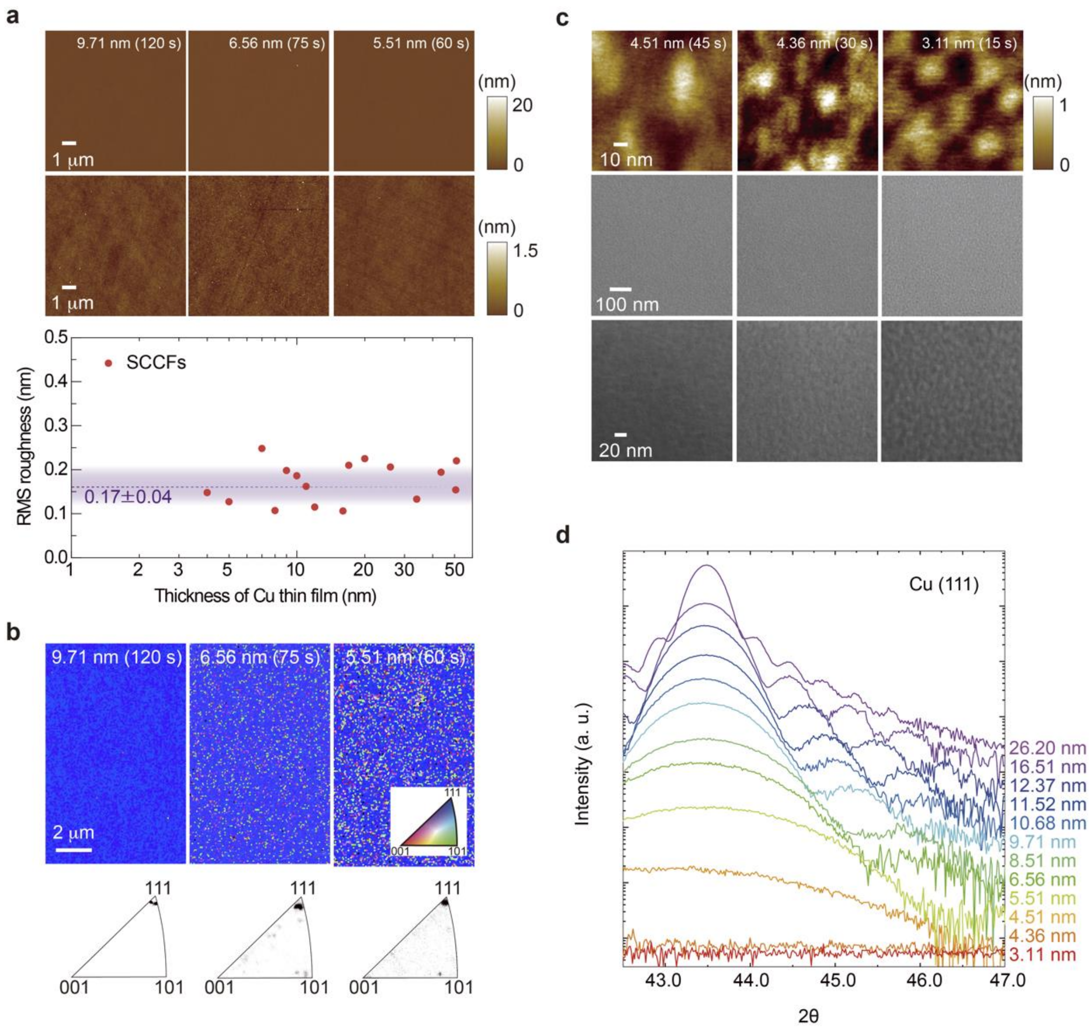

Supplementary Fig. 3. Structural characterisation using atomic force microscopy (AFM), X-ray diffraction (XRD) and electron backscatter diffraction (EBSD). a, (Top, first and second rows) AFM images of films with thickness > 5 nm (9.71, 6.56 and 5.51 nm). (Bottom) root-mean-square (RMS) roughness as a function of film thickness. The average RMS roughness of films with thickness < 50 nm (purple dashed line) was 0.17 ± 0.04 nm. **b**, (Top) EBSD images and (bottom) pole figures (PFs) of Cu thin films in stage II. **c**, (First row) AFM and (second and third rows) scanning electron microscopy images of Cu thin films in stage I with thickness < 5 nm (3.11, 4.36 and 4.51 nm). **d**, XRD spectra near the Cu(111) peak. Pendellösung oscillations were visible for thicknesses ranging from 6.56 to 26.20 nm, indicating that the Cu films have well-defined parallel surfaces.

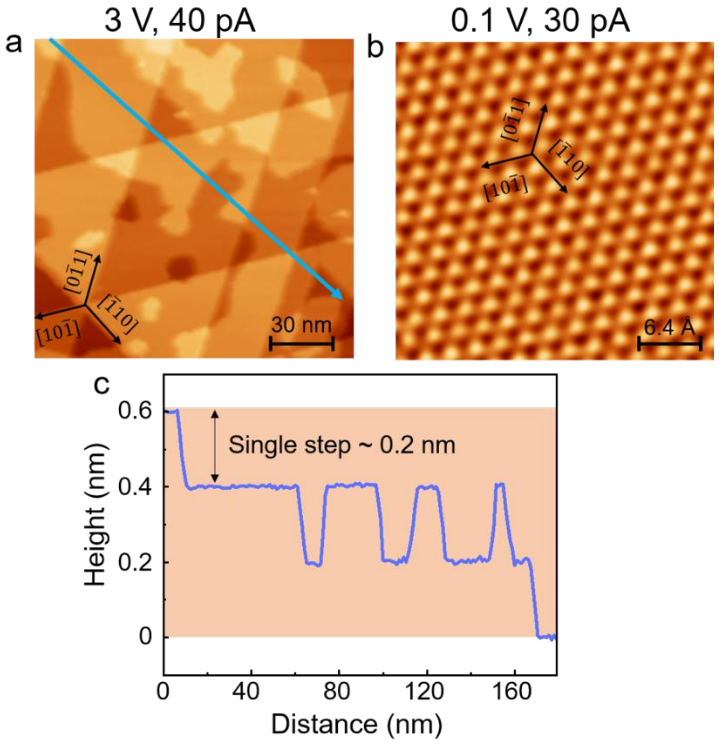

Supplementary Fig. 4. Scanning Tunneling Microscope (STM) topography images of the Cu(111) single-crystal film. a, Configuration of step edges for an imaging area of 150 nm × 150 nm. **b,** Atomic-resolution surface structure of 3 nm × 3 nm area. The arrows indicate the three corresponding orientations on the (111) plane. **c,** Profile of step height obtained from the line scan marked with a sky blue arrow in a, which shows a series of mono-atomic steps corresponding to the planar spacing of the Cu(111)

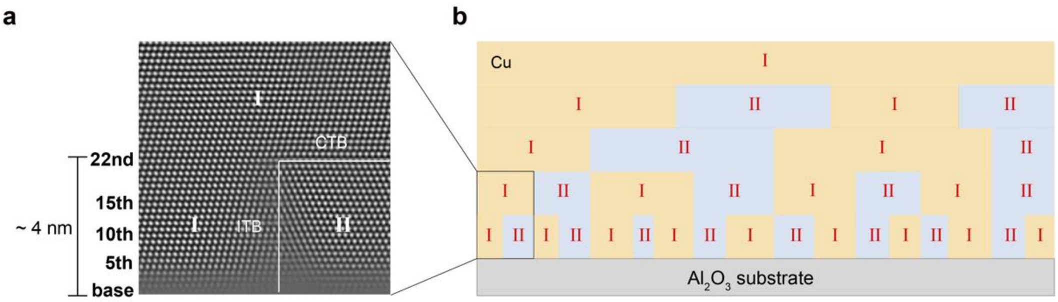

Supplementary Fig. 5. Merging of two orientations (ORs) according to thin-film thickness growth. a, High-resolution high-angle annular dark-field scanning transmission electron microscopy image near the twin boundary separating regions I and II with different stacking orders. ORs I and II merge with OR I at the 23rd layer. **b,** Schematic diagram of the merging process of ORs according to thin-film thickness growth.

Supplementary Note 1. Layers (23) of Cu(111) corresponding to ~5 nm in thickness and eventually thin film has almost a single orientation when the thickness reaches ~80 nm. Ideally, the trillions of nanodroplets nucleated near the interface are merged almost into one after reaching ~80 nm in thickness.

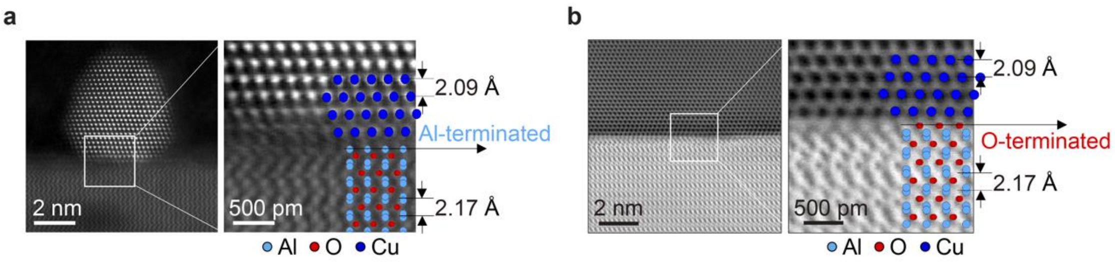

Supplementary Fig. 6. Transition of surface termination during growth using high-resolution scanning transmission electron microscopy (HRSTEM) image. a, Structures of the Cu nanodroplet/Al_2O_3. **b,** Cu film/Al_2O_3 interfaces with an orientation relationship of $(111)_{Cu}//(0001)_{Al2O3}$.

Supplementary Note 2. The high-resolution scanning transmission electron microscopy (HRSTEM) image of a Cu nanodroplet residing on an Al_2O_3 substrate shown in **a** is presented again for the analysis of the interfacial structure. Note that the HRSTEM imaging mode used for the Cu film/Al_2O_3 substrate interface was the ABF imaging mode. This mode shows inverted atomic contrast but can effectively collect weakly scattered signals from light elements, such as oxygen, in thicker samples. On the other hand, the medium-angle annular dark-field imaging mode of HRSTEM was used for imaging the interfacial structure between the thin Cu nanodroplet and the Al_2O_3 substrate.

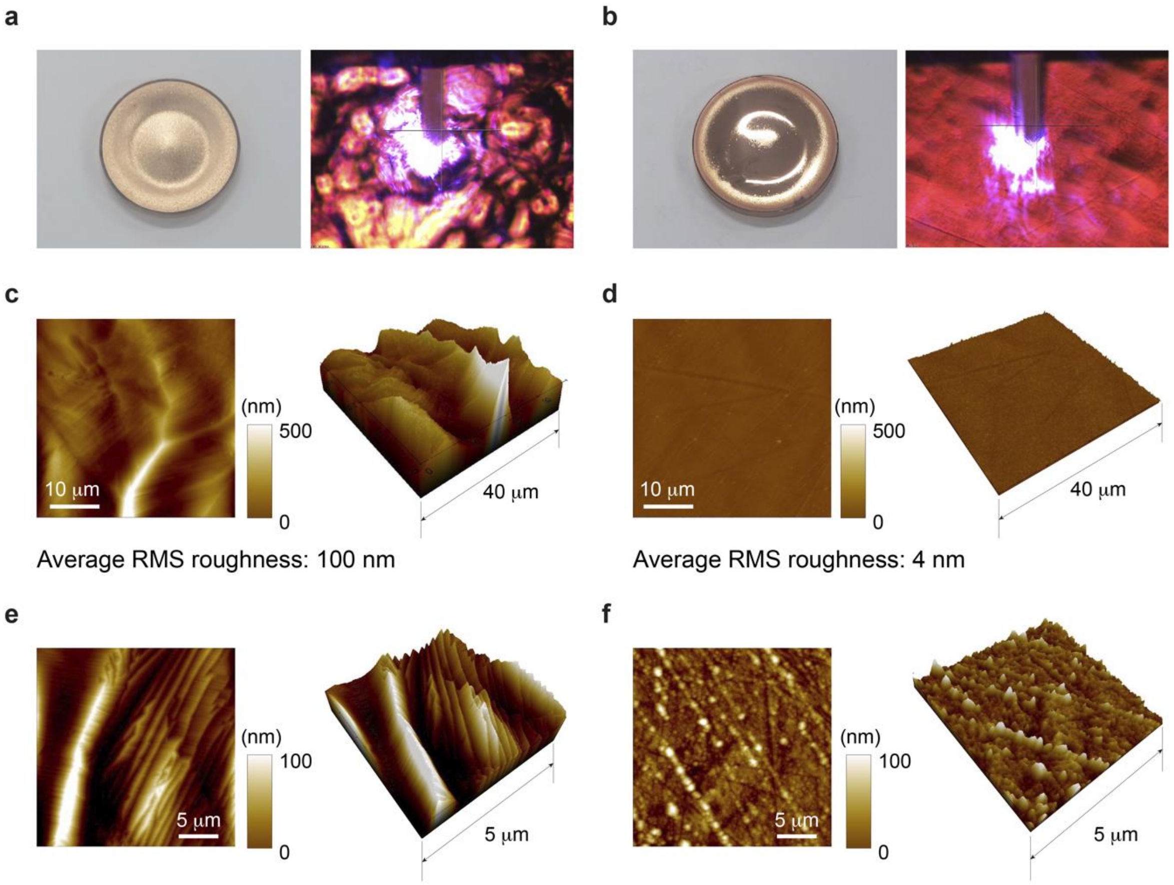

Supplementary Fig. 7. Difference in the surfaces of targets after deposition between the conventional sputtering method and atomic sputtering epitaxy (ASE). a, b, Photographs (left panel) and optical microscopy images (right panel) of the polycrystalline Cu target after sputtering using the conventional system (a) and of the single-crystal Cu target after the deposition using ASE (b). **c**, **d**, Surface roughness of **a** and **b** observed by atomic force microscopy (AFM) at the 40-μm scale in the lateral direction and 500-nm scale in the height direction. **e**, **f**, Surface roughness of **a** and **b** observed by AFM at the 5-μm scale in the lateral direction and at the 100-nm scale in the height direction.

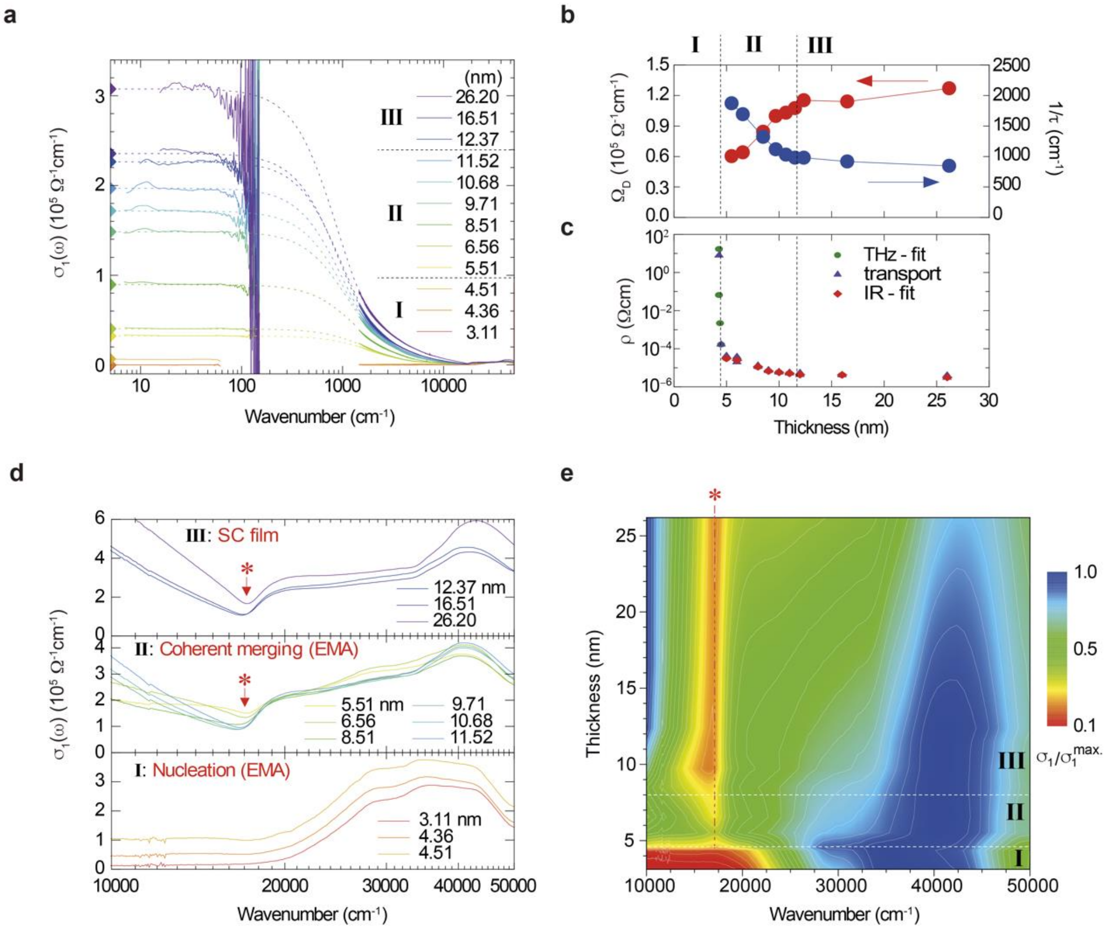

Supplementary Fig. 8. Terahertz (THz) and optical spectra of samples in the three stages. a, Measured optical conductivity spectra of 12 Cu film samples along with THz spectra below 100 cm^{-1} and Drude model fits (thin dashed lines). **b,** Thickness-dependent Drude fitting parameters including the plasma frequency (Ω_D) and impurity scattering rate ($1/\tau$). **c,** Comparison of direct current (DC) resistivity obtained using Drude fitting parameters and parameters directly measured using the DC transport technique and obtained from THz spectra. **d,** Near infrared-ultraviolet (NIR-UV) conductivity spectra above 10,000 cm^{-1} in the three stages. **e,** Colour-coded diagram of the NIR-UV conductivity spectra of all 12 Cu film samples. Thickness-dependent plasma-edge evolution (along the dot-dot-dashed line) supported three distinct growth stages.

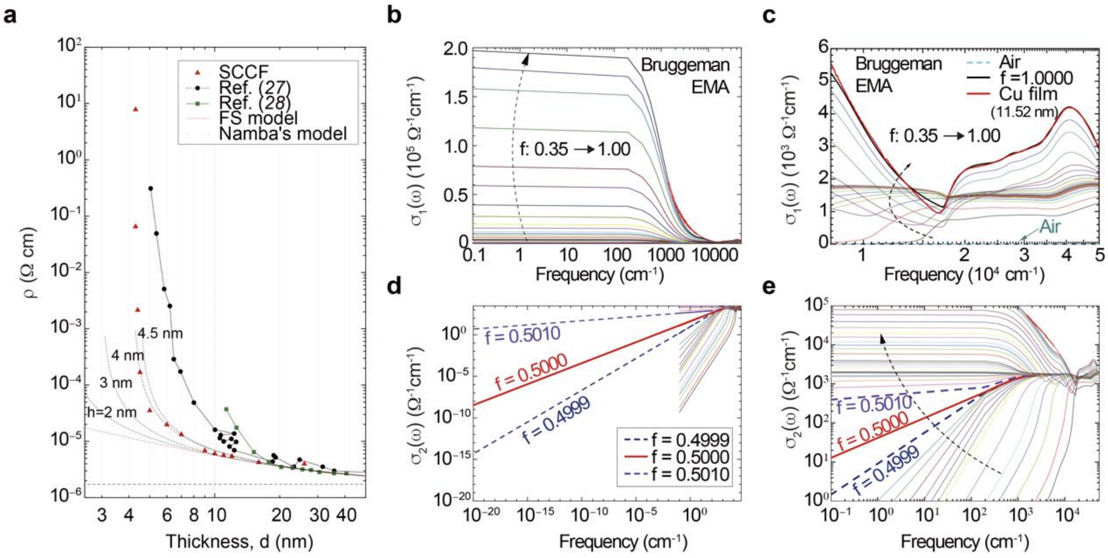

Supplementary Fig. 9. Conventional Fuchs–Sondheimer (FS) model fit and Namba model simulations and effective medium approximation (EMA) analysis. a, The Namba model matched our measured resistivity data well for a roughness amplitude of 4–4.5 nm, which was close to the size (height) of the Cu nanodroplets. However, the Namba model could not simulate the resistivity data (essentially infinite resistivity) in stage I with isolated Cu nanodroplets because this model was applicable only to the well-developed (or continuous) metallic thin films observed in stages II and III. The observed abrupt divergence in resistivity is not a common behaviour of (continuous) metallic thin films; therefore, another model is required to explain all of the measured resistivity (or conductivity) data, including the abrupt divergence in resistivity (or zero conductivity). Therefore, we used an EMA model to fully describe the entire thickness range, including stage I with isolated Cu nanodroplets, using a volume fraction parameter. **b**, Optical conductivity over a wide spectral range from 0.1 to 50,000 cm^{-1} was simulated using the EMA model at various volume fractions from 0.3500 to 1.000. **c,** Magnified view of the simulated EMA optical conductivity in a high-frequency region. The optical conductivity of air is exactly zero over the entire frequency region. **d**, Examples of extrapolations to near-zero frequency (10^{-20} cm^{-1}), which we used as the zero frequency for our DC conductivity estimates. **e,** Magnified view showing an abrupt slope change in EMA conductivity with respect to the frequency, which occurred at $f = 0.5$.

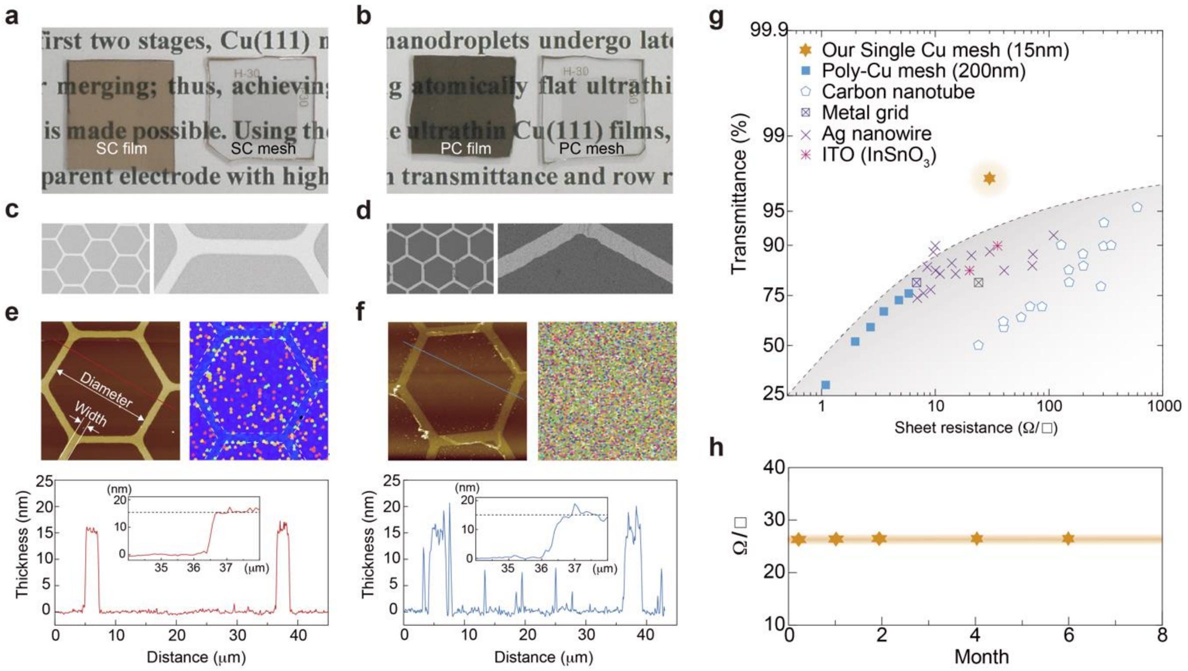

Supplementary Fig. 10. Transparent Cu mesh electrodes. **a**, A 15-nm-thick single-crystal (SC) Cu film and SC honeycomb mesh electrode on a sapphire substrate. **b**, A polycrystalline (PC) Cu film and PC honeycomb mesh electrode with a thickness of 15 nm deposited on a glass substrate. **c**, **d**, Scanning electron microscope images of (**c**) SC and (**d**) PC meshes. Enlarged images are also displayed. **e**, (Upper left) atomic force microscopy (AFM) image and (upper right) electron backscatter diffraction map of the SC mesh and (lower) its height profile along the indicated line, as obtained by AFM, along with (inset) a magnified view of part of the profile. **f**, Corresponding images and profile of the PC mesh. **g**, Comparison of the transmittance at 550 nm and sheet resistance of our SC mesh with those reported for other trichloroethylene materials, including carbon nanotubes, metal grids, Ag nanowires, and indium tin oxide.[34] **h**, Sheet resistances (Ω/sq) of freshly prepared and 1-, 2-, 4- and 6-month-old samples, which demonstrate the aging effect.

Supplementary Table 1. Atomic force microscopy thickness estimates.

Target thickness (nm)	Deposition time (s)	Thickness (nm)	Roughness (nm)
26	360	26.20	0.21
16	216	16.51	0.11
12	180	12.37	0.11
11	150	11.52	0.16
10	135	10.68	0.19
9	120	9.71	0.12
8	90	8.51	0.10
6	75	6.56	0.24
5	60	5.51	0.13
4	45	4.51	0.15
3	30	4.36	0.13
2	15	3.11	0.13

Supplementary Table 2. Comparison of Al_2O_3/Cu(111) interfaces.

Al_2O_3 surface	Surface energy ($J\ m^{-2}$)	Diffusion barrier for Cu atoms (eV)	Interfacial energy ($J\ m^{-2}$)	Adhesion energy ($J\ m^{-2}$)
Al-terminated	1.59	0.23	−24.51	0.68
O-terminated	4.26	1.44	−25.95	4.81
Difference	2.67	1.21	−1.44	4.10
